# Prolonged Taping with Exercise Therapy for Patellofemoral Pain in Adults: A Systematic Review and Single-Arm Meta-Analysis

**DOI:** 10.3390/jcm13237476

**Published:** 2024-12-09

**Authors:** Christian A. Than, Maamoun Adra, Tom J. Curtis, Yasmine J. Khair, Hugh Milchem, Sum-Yu C. Lee, Goktug Şanli, Karen Smayra, Hayato Nakanishi, Zaher Dannawi, Belinda R. Beck

**Affiliations:** 1 Faculty of Medicine, St. George’s University of London, London SW17 0RE, UK; 2 University of Nicosia Medical School, University of Nicosia, 2417 Nicosia, Cyprus; 3 School of Biomedical Sciences, The University of Queensland, St. Lucia, Brisbane, QLD 4072, Australia; 4 Frimley Health NHS Foundation Trust, Frimley GU16 7UJ, UK; 5 Faculty of Sports Sciences, Marmara University, 34722 Istanbul, Turkey; 6 Mid and South Essex NHS Foundation Trust, Essex SS0 0RY, UK; 7 School of Health Sciences and Social Work, Menzies Health Institute, Gold Coast, QLD 4222, Australia; b.beck@griffith.edu.au; 8 The Bone Clinic Pty Ltd., 26 Turbo Drive, Coorparoo, QLD 4151, Australia

**Keywords:** patellofemoral pain, taping, exercise, McConnell, Kinesio

## Abstract

**Purpose**: To investigate the effects of prolonged taping on patellofemoral pain (PFP). **Methods**: A literature search of PubMed, EMBASE (Elsevier), CiNAHL, Cochrane Central Register of Controlled Trials, Cochrane Database of Systematic Reviews, Scopus, and Web of Science was conducted from database inception to 28 June 2024. Eligible studies reported PFP patients over 18 years of age undergoing an exercise protocol with additional taping that was maintained outside of exercise sessions (PROSPERO ID: CRD42023422792). **Results**: Seventeen studies met the eligibility criteria with 348 patients (*n* = 221 Kinesio taping, McConnell taping *n* = 127). For pain, the Kinesio baseline scores were 5.73 (95% CI: 4.73–6.73, I^2^= 97%), and the McConnell scores were 5.05 (95% CI: 3.82–6.28, I^2^ = 95%). At the combined recent follow-up, the Kinesio scores were 2.14 (95% CI: 1.11–3.18, I^2^ = 98%), and the McConnell scores were 2.58 (95% CI: 0.79–4.37, I^2^ = 98%). For functionality, the Kinesio baseline scores were 64.19 (95% CI: 53.70–74.68, I^2^ = 98%), and the McConnell scores were 68.02 (95% CI: 65.76–70.28, I^2^ = 0%). At the combined recent follow-up, the Kinesio scores were 84.23 (95% CI: 79.44–89.01, I^2^ = 95%), and the McConnell scores were 86.00 (95% CI: 83.82–88.17, I^2^ = 0%). The minimum clinically important difference (MCID) was achieved for both modalities at 6 weeks and beyond. **Conclusions**: Prolonged taping that remains on PFP patients outside of isolated exercise sessions appears beneficial in reducing pain and increasing functionality.

## 1. Introduction

Patellofemoral pain (PFP) describes anterior or retro patellar pain that is aggravated by activities that load the patella during weight-bearing on a flexed knee [1]. It is often used synonymously with other terms such as anterior knee pain, runner’s knee, or patellofemoral syndrome despite the diagnostic difficulties created with improper interchanging nomenclature [2,3]. A documented gender disparity exists in diagnoses, with a twofold higher annual incidence in women [3]. Currently, it is thought to account for 7.3% of all diagnoses for patients seeking orthopedic medical care within the US [3]. Furthermore, the morbidity caused by PFP has been shown to directly lead to increased healthcare costs from nonoperative and operative treatment therapies and indirectly from decreased patient productivity [4].

Numerous management modalities exist for PFP, with physical therapy being the conservative gold standard to decrease pain and increase function [5,6]. However, no consensus exists on the most effective physical therapy modalities or specific treatment protocols to employ [5]. In a bid to find the optimal management approach, various supplementations to exercise regimes have been examined [5]. Taping has gained popularity with treating physiotherapists from its relatively low expense and quick application, alongside a documented reduction of pain and an improvement in patient knee function [7,8,9]. The exact mechanism of the improvement remains unknown, but the prevailing theory involves the realigning of the patella and the unloading of soft tissues to reduce pain [10]. The two major variants of patellar taping include the McConnell taping and Kinesio taping techniques. The former regulates mediolateral forces on the patella for improved joint alignment, while the latter aims to manage vastus medialis oblique and vastus lateralis muscle imbalance [11]. Both are regarded as viable methods to improve muscle activity, motor function, and patient quality of life [11]. Regardless of the taping technique or specific application method used on the lower limb, the primary goal remains consistent in attempting to alleviate PFP.

At present, while improvements in pain and function have been reported, controversy still exists regarding the clinical significance of improvement with taping [12]. A reason for this could be from a lack of consensus on a standardized protocol surrounding taping parameters. Variations in tape type, direction of pull on the patella, or application to musculature, as well as number of layers applied are all variables left to the discretion of the treating team and patient [9]. One taping parameter that has not been properly assessed is the duration for which the tape is applied, be that only during treatment sessions or left on during waking hours [9]. Earlier meta-analyses have investigated the overall effects of taping with variations in the length of time for tape application [9,11,13]. However, these studies did not isolate the effects of using exercise in combination with supplemental taping alone. Furthermore, no study to date has exclusively investigated the effects of prolonged taping applied outside of standard-of-care physical therapy sessions. Hence, the aim of this systematic review and single-arm meta-analysis is to examine the effects of prolonged taping for PFP, continuously remaining on patients outside of isolated exercise intervention sessions, on pain and function.

## 2. Methods

### 2.1. Search Strategy and Data Sources

This review followed the Preferred Reporting Items for Systematic Reviews and Meta-analyses (PRISMA) guidelines [14]. A comprehensive search of several databases from each database’s inception to 28 June 2024 was conducted with no language restriction. The databases included PubMed, EMBASE (Elsevier), CiNAHL, Cochrane Central Register of Controlled Trials, Cochrane Database of Systematic Reviews, Scopus, and Web of Science. The Cochrane Database of Systematic Reviews was included to hand search the references of any relevant prior literature. The search strategy was designed and conducted by a medical reference librarian. Controlled vocabulary supplemented with keywords was used to search for studies describing patellofemoral pain or anterior knee pain with taping or strapping. The actual strategy listing all search terms used and how they are combined is available in Supplementary Materials File S1. This review was registered prospectively with PROSPERO (CRD42023422792).

### 2.2. Eligibility Criteria and Quality Assessment

Eligible studies must have met all the following inclusion criteria: (1) patients over 18 years of age with PFP; (2) have undergone therapeutic intervention through patellofemoral taping with either Kinesio or McConnell taping; (3) have undergone an exercise protocol in addition to taping; (4) report on the primary outcomes of visual analog scale (VAS), numerical rating score (NRS) or anterior knee pain scale (AKPS) (also known as Kujala score); (5) employed a minimum 2-week intervention period; (6) employed a randomized, comparative, case-control, prospective, or retrospective observational cohort study design. Exclusion criteria were (1) employed treating session was only taping (i.e., only for the day of the exercise protocol or shorter); (2) employment of any other therapies besides taping and exercise (e.g., heat packs, massage, transcutaneous electrical nerve stimulation, mobilization, anti-inflammatory medications); (3) patients with prior knee surgery; (4) existence of other pathological diagnoses outside of PFP (e.g., osteoarthritis, osteochondritis dissecans); (5) employed a case report or case series design or were abstracts or conference presentations; (6) any studies with overlapping patient data; (6) any articles not translatable to English.

Article screening and data extraction were conducted by three independent assessors (MA, TJC, SCL). Any disagreements were adjudicated by CAT and discussed with co-authors as necessary. The quality of each study was assessed by two independent authors (YJK and HM) using version 2 of the Cochrane risk-of-bias tool for randomized trials (ROB2) [15] and risk of bias in non-randomized studies of interventions ROBINS-I [16]. In cases of disparity, two independent assessors deliberated, and disagreements were settled through adjudication by CAT.

### 2.3. Outcomes

Pain and function were the primary outcome variables of interest in this meta-analysis. Patient perception of pain was evaluated using VAS or NRS questionnaires. A lower VAS or NRS score indicated lower perceived pain, meaning greater patient relief. For analysis, VAS and NRS scores were combined where NRS scores were on a measure of 0–100 points [17,18]. Where VAS was only reported during activity, the least strenuous VAS activity was combined with non-activity VAS and NRS of other studies. Patient perception of functionality was evaluated using AKPS scores. A higher AKPS score indicated less disability, meaning more patient functionality.

### 2.4. Data Extraction and Minimal Clinical Important Difference (MCID) Interpretation

The VAS, NRS, and AKPS scores were extracted in the following epochs: baseline (pre-intervention), 2 weeks, 4 weeks, 6 weeks, and 12 weeks post-intervention as well as combined for the final follow-up across included studies. A VAS or NRS score decrease of 2 points was applied as the minimum clinically important difference (MCID) in patients with PFP as previously reported [19]. An AKPS score increase of 8 points was applied as the MCID in patients with PFP, as previously reported [19].

### 2.5. Statistical Analysis

Means of continuous variables and rates of binary variables were pooled using the generic inverse variance method of DerSimonian, Laird [20]. Proportions underwent logit transformation prior to meta-analysis. The heterogeneity of effect size estimates across the studies was quantified using the Q statistic and the I^2^ index (*p* < 0.10 was considered significant) [21]. A value of I^2^ of 0–25% indicates minimal heterogeneity, 26–50% moderate heterogeneity, and 51–100% substantial heterogeneity. The random-effects model was used [21]. If mean and standard deviation (SD) were unavailable, median was converted to mean using the formulas from the Cochrane Handbook for Systematic Reviews of Interventions [22]. If SD was not available or extractable, the reported mean was omitted from the calculation. Authors were contacted three times to obtain any relevant additional information that was omitted in published articles. Publication bias was assessed visually using funnel plots [23]. Data analysis was performed using Open Meta analyst software v0.24.1 (CEBM, Brown University, Providence, RI, USA). All included studies were categorized as having one study arm for analysis. Included studies that involved multiple arms had only the arms of relevance extracted (i.e., taping with exercise only). For the purpose of the one-arm meta-analysis, studies that had multiple eligible study arms for extraction (e.g., one-arm Kinesio taping and a separate arm of McConnell taping) were all included and treated separately during analysis.

## 3. Results

**Figure 1:**
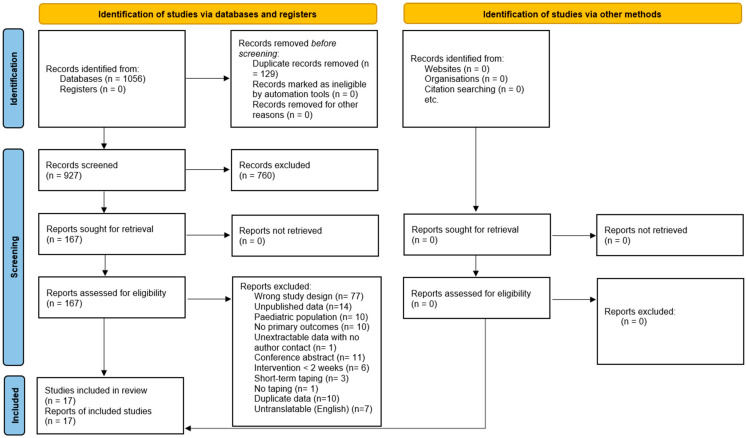
Preferred reporting items for systematic reviews and meta-analyses (PRISMA) flowchart.

The initial search yielded 927 potentially relevant articles from which seventeen unique studies, involving 348 patients (*n* = 221 Kinesio taping, McConnell taping *n* = 127), met the eligibility criteria [24,25,26,27,28,29,30,31,32,33,34,35,36,37,38,39,40]. The PRISMA flowchart illustrates the details of the study selection process in Figure 1. Of the articles reporting on patient gender, 24.42% (*n* = 74) were male and 75.57% (*n* = 229) were female [24,25,26,27,28,29,32,33,34,35,36,37,38,39,40]. Twelve studies were randomized control trials [24,25,26,27,28,30,31,32,33,36,37,39]. Three studies were non-randomized clinical trials [29,34,38]. One study was a retrospective comparative study [35]. One study was a prospective cohort study [40]. Eleven studies used Kinesio taping interventions [24,25,26,28,29,32,34,35,36,37,40]. Five studies used McConnell taping interventions [27,30,31,33,39]. One study used both Kinesio and McConnell taping interventions in separate arms of the study [38]. Two studies compared taping with open chain exercise against taping with closed chain exercise [30,36]. Of the articles reporting on patient age, the mean age range was from 25.00 years to 57.97 years [24,25,26,27,28,31,32,33,34,35,36,37,38,39,40]. The baseline characteristics of each included study are described in Table 1.

### 3.1. Risk of Bias

**Figure 2:**
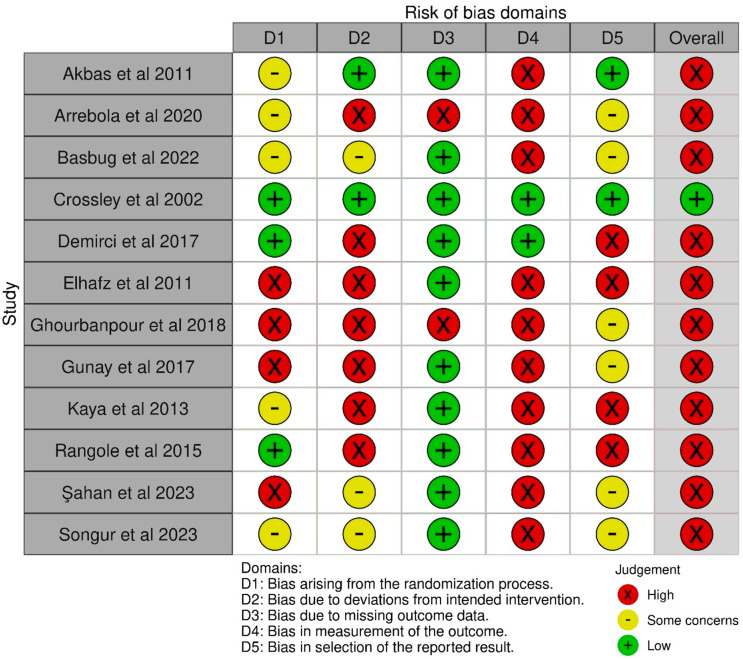
Version 2 of the Cochrane risk-of-bias tool for randomized trials (ROB2) assessment [24,25,26,27,28,30,31,32,33,36,37,39].

**Figure 3:**
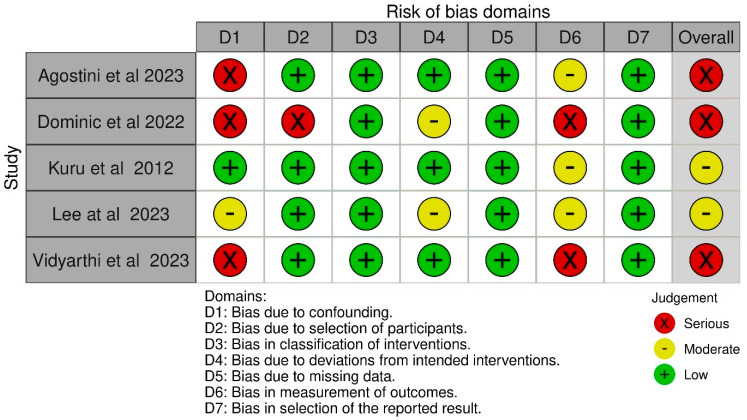
Risk of bias in non-randomized studies of interventions (ROBINS-I) assessment [29,34,35,38,40].

The results of the quality assessment of all included studies are shown in Figure 2 and Figure 3. Of the randomized control trials judged via the ROB2 Tool, one was found to be of low risk [27]. Eleven were found to be of high risk [24,25,26,28,30,31,32,33,36,37,39]. High-risk concerns were noted in domains of the randomization process [30,31,32,37], deviations from the intended intervention [25,28,30,31,32,33,36], missing outcome data [25,31], the measurement of the outcome [24,25,26,30,31,32,33,36,37,39], and the selection of the reported result [28,30,33,36]. Of the observational studies judged via the ROBINS-I tool, two were found to be of moderate risk [34,35]. Three were found to be of serious risk [29,38,40]. Serious risk was noted in the domains of confounding [29,38,40], the selection of participants [29], and in the measurement of outcomes [29,40].

### 3.2. Taping and Exercise Modality

The duration the tape was kept on patients, outside of exercise sessions, ranged from renewal daily [27,33,35,38], renewal weekly [26], renewal twice weekly [28,32], renewal thrice weekly [30,31], renewal every 2–5 days [29,34,36,38,39], maintenance intervals of 3–5 days [24,25], maintenance intervals of 6 days [40], and unspecified continuous application [37].

All studies employed a general exercise program involving the flexors and or extensors of the knee, with varying sets, repetitions, session length, involvement of other muscle groups, and stretching. Exercise session frequency ranged from daily [27,28,33,34,36,37,38,39], twice daily [35], once weekly [26], twice weekly [25,32], thrice weekly [29,30,31], and unspecified [24,40]. The taping and exercise modalities of each included study are described in Table 1**.** The protocol for taping and exercise for each included study is summarized in Supplementary Table S1.

### 3.3. Visual Analog Scale (VAS) and Numerical Rating Scale (NRS) Scores

Pain scores at baseline and for each follow-up point are summarised in Table 2. Twelve studies reported on VAS during no activity [24,27,28,29,30,31,32,34,36,37,39,40]. Two studies reported on VAS only during activity from which the least strenuous activities of descending stairs [26] and step down [33] were taken. Two studies reported NRS during no activity [25,38].

The included studies using Kinesio taping allowed pooling at epochs for 2 weeks, 4 weeks, 6 weeks, and 12 weeks [24,25,26,28,29,32,34,36,37,38,40]. The Kinesio taping pain score reduction from baseline was −2.73 points at 2 weeks, −2.55 points at 4 weeks, −3.98 points at 6 weeks, and −4.28 points at 12 weeks. When combining the most recent follow-up of each included study, the mean reduction from baseline was −3.59 points. MCID was achieved at all time points due to a decrease of 2 points [19]. The forest plot of each Kinesio taping intervention pain score time point is available in Supplementary Figure S1.

The included studies using McConnell taping allowed pooling at epochs for 4 weeks and 6 weeks [27,30,31,33,38,39]. The McConnell taping pain score reduction from baseline was −0.66 points at 4 weeks and −4.48 points at 6 weeks. When combining the most recent follow-up of each included study, the mean reduction from baseline was −2.47 points. MCID was achieved at 6 weeks and when combining all final follow-up points due to a decrease of 2 points [19]. The forest plots of each McConnell taping intervention pain score time point are available in Supplementary Figure S2. The inclusion of less than 10 studies at each follow-up point for both Kinesio and McConnell taping interventions limited distinguishing publication bias, via funnel plot inspection, due to chance from real asymmetry.

### 3.4. Anterior Knee Pain Score (AKPS)

Functional scores at baseline and for each follow-up point are summarized in Table 3. The included studies using Kinesio taping allowed pooling at epochs for 2 weeks and 6 weeks [24,25,28,32,34,35,36,37]. The Kinesio taping functional score increase from baseline was 22.02 points at 2 weeks and was 18.61 points at 6 weeks. When combining the most recent follow-up of each included study, the mean increase from baseline was 20.04 points. MCID was achieved at all time points due to an increase of 8 points [19]. The forest plot of each Kinesio taping intervention functional score time point is available in Supplementary Figure S3.

The included studies using McConnell taping allowed pooling at epochs for 6 weeks only [27,39]. The McConnell taping functional score increase from baseline to combining the most recent follow-up of 6 weeks was 17.98 points. MCID was achieved at all time points due to an increase of 8 points [19]. The forest plot of each McConnell taping intervention functional score time point is available in Supplementary Figure S4. The inclusion of less than 10 studies at each follow-up point for both Kinesio and McConnell taping interventions limited distinguishing publication bias, via funnel plot inspection, due to chance from real asymmetry.

## 4. Discussion

The efficacy of taping in PFP beyond treatment sessions or short-term applications (in addition to usual care exercise therapy) was unknown. The current study is the first meta-analysis to examine existing evidence of prolonged taping duration on PFP and the quality of that evidence. Our findings suggest that exercise with supplemental prolonged taping using either Kinesio or McConnell techniques will improve pain and functionality as indicated by VAS, NRS, and AKPS score improvement. Both taping modalities achieve a clinically important difference at 6 weeks post-intervention or further. These findings provide support for the use of tape remaining on patients outside of isolated treatment sessions should the patient or treating team desire. The results presented here establish a framework for future randomized trials to explore varying taping lengths, aiming to identify the optimal approach for managing PFP. They also provide a rationale for conducting comparator studies against exercise-only interventions to clarify the true impact that supplemental taping may have.

Many pathophysiological theories exist as to the cause of PFP. Common to most are physiological mechanisms such as patellar multitracking and dynamic valgus (functional malalignment), due to weak hip abductors or abnormal rear-foot eversion with pes pronatus valgus [41]. Other contributions are attributed to vastus medialis/vastus lateralis imbalance or iliotibial band tightness [41]. Overall, dysfunction in the extensor mechanism seems to be the greatest contributing factor in PFP [32]. The exact cause of pain in PFP is unclear, which challenges the determination of definitive treatment modalities [35].

The general goal of treatment is to restore the biomechanics of the joint and increase vastus medialis oblique strength to decrease retraction and stress to the patellofemoral joint. Conservative treatment is the mainstay through exercise therapy to reduce the aforementioned retraction and stress on the joint [42]. However, clinical studies support the positive benefits of taping in helping to correct patellar malalignment and/or facilitate vastus medialis oblique contractions which can help reduce pain [43]. Particularly, tape that is applied medially has some potential to correct lateral patellar maltracking and tilt, with effective reduction in symptoms [44]. Hypothetically, tape that remains applied to the knee could thus continually exert this force for a combined synergistic effect with exercise on pain and function. The results of this meta-analysis provide some evidence for this theory, as demonstrated by the improvements in pain and functionality. From this viewpoint, prolonged taping with exercise does have clinical relevance, as demonstrated by the achievement of MCID in outcomes of pain and functionality.

The Kinesio and McConnell taping mechanisms differ significantly, affecting their preferred use by clinicians [11]. Kinesio taping employs an elastic, flexible tape designed to facilitate muscle function, increase proprioception, and improve circulation by mimicking the elastic qualities of the skin [8]. In contrast, McConnell taping utilizes a rigid, inelastic tape purported to improve patellar tracking, thus reducing joint friction and mechanical stress [44]. The main difference between these taping methods lies in their elasticity and intended function. Kinesio taping is less restrictive than McConnell taping; however, it does not have a significant impact on patellar alignment. Our analysis revealed that both techniques showed clinically significant improvements in pain perception and function at six weeks, suggesting that the choice between them should depend on the patient’s specific needs and the desired outcome. Kinesio taping could be a better option for providing continuous support, whereas McConnell taping might be more advantageous for promptly correcting mechanical issues during specific activities. Consequently, Kinesio taping may be preferred for daily support and ongoing muscle facilitation, whereas McConnell taping is best applied for acute correction of patellar alignment during exercise. This tailored approach ensures that clinicians choose the most appropriate taping method based on the patient’s needs and the specific clinical scenario. Nevertheless, future randomized control trials should be conducted to compare modalities.

The current study exclusively focused on adult participants to avoid the developmental differences that can significantly influence treatment outcomes in adolescents. Previous meta-analyses often included mixed-age populations, which introduced heterogeneity due to physiological and biomechanical variances between adolescents and adults [9,13,43]. For instance, the meta-analysis by Callaghan and Selfe (2012) highlighted the potential divergence in the mechanism of intervention between adolescents and older populations due to the ongoing maturation of their musculoskeletal system [9]. By limiting the scope to adults, this study ensured a more homogeneous sample, leading to potentially more reliable conclusions regarding the efficacy of prolonged Kinesio and McConnell taping on PFP. This approach aligns with recommendations for targeted research designs to accurately assess treatment efficacy in specific demographics [21]. Future research should continue to stratify participants by age to avoid potential biases and ensure that interventions are appropriately tailored to each group’s unique physiological needs.

The current meta-analysis underscores the need for higher-quality studies to compare the effectiveness of exercise-only interventions versus combined taping and exercise protocols for PFP. Previous studies have predominantly focused on the short-term effects of taping, demonstrating immediate pain relief and functional improvements [8,45]. However, the lack of robust longitudinal studies and comprehensive meta-analyses addressing long-term outcomes and direct comparisons between short-term and long-term taping presents a significant gap in the literature. This gap highlights the importance of future research to explore these areas to better understand the sustained benefits of these interventions. Additionally, no comprehensive meta-analysis including only adults has yet to adequately address the comparison between exercise alone and the combination of exercise with taping, mainly due to insufficient participant numbers and inconsistencies in study methodologies. This inadequacy in participant numbers significantly affects the statistical power and the ability to draw definitive conclusions.

The heterogeneity seen within our outcomes across studies may primarily be due to variations in exercise protocols, tape application techniques, and follow-up periods [9]. This inconsistency complicates the synthesis of data and the formulation of generalized recommendations. To address this, future studies should implement standardized exercise protocols and clearly defined follow-up points. These measures will help reduce variability and improve the reliability of findings, enabling a more accurate assessment of taping’s therapeutic benefits. Overall, while taping shows promise in managing PFP, more well-designed, large-scale studies are necessary to establish its efficacy compared to exercise alone and clarify the differential effects of short-term and long-term taping.

## 5. Limitations

As with all meta-analyses, limitations are present within the current study. Foremost is the high heterogeneity in outcomes, suggesting a cautionary approach to the interpretation of results. This also prohibits discussion on whether any protocol is more effective than another at improving pain and function in the patient population. Furthermore, the high risk of bias seen across the included articles presents a substantial reduction in the strength of evidence found within the current meta-analysis for drawn conclusions. Second, all the included studies were completed in a relatively short period of time, which does not give a conclusive outlook as to the long-term significance of taping. Third, although some studies reported monitoring exercise compliance and or taping adherence, not all studies employed thorough compliance protocols. Fourth, for ethical reasons, patients could not be randomized to further supplemental treatments such as analgesics or undocumented massage therapy, and a lack of adequate reporting prevented controlling for their effects. Fifth, inadequate reporting on outcomes such as changes in observable functionality (e.g., single-jump hop tests or 10-step stair climbing tests) or biomechanical measures (e.g., peak torque) precluded any elucidation on the effect taping and exercise may have had on such parameters. Leading from this, VAS and NRS scales were combined due to prior literature on their correlation and the unavailability of reporting within current studies to allow a separate analysis. Finally, not all studies controlled for physiotherapist experience in application of the taping technique, or may have relied on patient self-application, which may have influenced outcomes.

## 6. Conclusions

The current work is the first meta-analysis to examine the effect of prolonged taping for patients with PFP. The results here demonstrate that taping with either Kinesio or McConnell modalities, combined with exercise, improves patient perception of pain and function as measured by VAS or NRS and AKPS, respectively. Thus, some clinical advantage appears present in using extended taping periods with the tape remaining on patients outside of exercise sessions. Further studies are required with higher sample sizes, standardized taping protocols, and longer follow-up times to elucidate the findings of this study. Furthermore, future randomized control trials are required to investigate the true effect of supplemental taping from exercise alone.

## Figures and Tables

**Table 1:** Baseline Characteristics.

Study	Country	Design	Taping Type	Sample Size (*n*)	Males: Females (*n*)	Mean Age ± SD (years)	Mean BMI ± SD (kg/m^2^)	Taping Length	Exercise Frequency	Follow-Up
Akbas et al., 2011 [24]	Turkey	RCT	Kinesio	15	0:15	41.00 ± 11.26	25.17 ± 4.80	5-day application interval for 6 weeks	NR	Baseline, 3 weeks, 6 weeks
Agostini et al., 2023 (Kinesio) [38]	Italy	Non-randomised clinical trial	Kinesio	16	4:12	30.40 ± 11.70	NR	Renewed every 3–4 days for 12 weeks	Daily	Baseline, 12 weeks
Agostini et al., 2023 (McConnell) [38]	Italy	Non-randomised clinical trial	McConnell	19	5: 14	30.79 ± 13.14	NR	Renewed daily for 12 weeks	Daily	Baseline, 12 weeks
Arrebola et al., 2020 [25]	Brazil	RCT	Kinesio	13	0:13	30.38 ± 8.40	24.37 ± 2.60	3 to 5-day application interval for 12 weeks	2× weekly	Baseline, 6 weeks, 12 weeks, 24 weeks
Basbug et al., 2022 [26]	Turkey	RCT	Kinesio	15	0:15	34.10 ± 8.90	23.90 ± 5.10	Renewed weekly for 6 weeks	Weekly	Baseline, 6 weeks, 12 weeks
Crossley et al., 2002 [27]	Australia	RCT	McConnell	36	13:23	29.00 ± 8.00	23.50 ± 3.80	Renewed daily for 6 weeks	Daily	Baseline, 6 weeks
Demirci et al., 2017 [28]	Turkey	RCT	Kinesio	17	0:17	36.70 ± 7.80	24.70 ± 4.90	Renewed 2× weekly for 2 weeks	Daily	Baseline, 2 weeks, 6 weeks
Dominic et al., 2022 [29]	India	Non-randomised clinical trial	Kinesio	15	6:9	NR	NR	Renewed every 3 days for 4 weeks	3× weekly	Baseline, 4 weeks
Elhafz et al., 2011 (open kinetic chain exercise group) [30]	Egypt	RCT	McConnell	15	NR	NR	NR	Renewed 3× weekly for 4 weeks	3× weekly	Baseline, 4 weeks
Elhafz et al., 2011 (closed kinetic chain exercise group) [30]	Egypt	RCT	McConnell	15	NR	NR	NR	Renewed 3× weekly for 4 weeks	3× weekly	Baseline, 4 weeks
Ghourbanpour et al., 2018 [31]	Iran	RCT	McConnell	15	NR	33.85 ± 10.29	24.70 ± 6.76	Renewed 3× weekly for 4 weeks	3× weekly	Baseline, 4 weeks
Günay et al., 2017 [32]	Turkey	RCT	Kinesio	16	5:11	36.00 ± 8.00	25.60 ± 2.60	Renewed 2× weekly for 6 weeks	2× weekly	Baseline, 6 weeks
Kaya et al., 2013 [33]	Turkey	RCT	McConnell	15	0:15	39.50 ± 12.40	NR	Renewed daily for 6 weeks	Daily	Baseline, 6 weeks
Kuru et al., 2012 [34]	Turkey	Non-randomised clinical trial	Kinesio	15	3:12	32.93 ± 12.17	23.65 ± 4.59	Renewed every 5 days for 6 weeks	Daily	Baseline, 6 weeks
Lee et al., 2023 [35]	Korea	Retrospective comparative study	Kinesio	20	5:15	27.50 ± 5.40	21.90 ± 2.30	Renewed daily for 4 weeks	2× daily	Baseline, 4 weeks
Rangole et al., 2015 (open kinetic chain exercise group) [36]	India	RCT	Kinesio	15	8:7	47.93 ± 5.94	NR	Renewed every 2–3 days for 2 weeks	Daily	Baseline, 2 weeks
Rangole et al., 2015 (closed kinetic chain exercise group) [36]	India	RCT	Kinesio	15	8:7	52.67 ± 5.88	NR	Renewed every 2–3 days for 2 weeks	Daily	Baseline, 2 weeks
Şahan et al., 2023 [37]	Turkey	RCT	Kinesio	14	2:12	25.00 ± 6.23	24.58 ± 3.66	Continuous for 6 weeks	Daily	Baseline, 6 weeks
Songur et al., 2023 [39]	Turkey	RCT	McConnell	12	3:9	28.00 ± 12.22	23.53 ± 3.55	Renewed every 3 days for 6 weeks	Daily	Baseline, 6 weeks
Vidyarth et al., 2023 [40]	India	Prospective cohort study	Kinesio	35	12:23	57.97 ± 4.96	NR	6-day application interval for 4 weeks	NR	Baseline, 2 weeks, 3 weeks, 4 weeks

N.B Agostini et al., 2023 are the same study split into Kinesio and McConnell taping groups. Elhafz et al., 2011 are the same study split into open kinetic chain and closed kinetic chain exercise groups. Rangole et al., 2015 are the same study split into open kinetic chain and closed kinetic chain exercise groups. Therefore, the description of 20 studies exists within this table. Overall, 17 unique separate studies were included in this meta-analysis. BMI: body mass index, NR: not reported, RCT: randomized control trial, SD: standard deviation.

**Table 2:** Pain Scores.

Outcomes	Mean	95% CI	I^2^	Included Study Groups (N)	Sample Size (N)	MCID Achieved
**Kinesio Taping**						
Baseline [24,25,26,28,29,32,34,36,37,38,40]	5.73	4.73–6.73	97%	12	201	NA
Follow-up at 2 weeks [28,36,40]	3.00	0.61–5.39	99%	4	82	Yes
Follow-up at 4 weeks [29,40]	3.18	0.62–5.73	99%	2	50	Yes
Follow-up at 6 weeks [24,25,26,28,32,34,37]	1.74	0.89–2.58	90%	7	105	Yes
Follow-up at 12 weeks [25,26,38]	1.45	0.04 2.86	96%	3	44	Yes
Combined recent follow-up [24,25,26,28,29,32,34,36,37,38,40]	2.14	1.11–3.18	98%	12	201	Yes
**McConnell Taping**						
Baseline [27,30,31,33,38,39]	5.05	3.82–6.28	95%	7	127	NA
Follow-up at 4 weeks [30,31]	4.39	2.91–5.88	93%	3	45	No
Follow-up at 6 weeks [27,33,39]	0.57	0.20–0.94	53%	3	63	Yes
Combined recent follow-up [27,30,31,33,38,39]	2.58	0.79–4.37	98%	7	127	Yes

N.B Studies [25,38] used the NRS whilst all other studies used the VAS. An MCID of 2 points decreased from baseline was accepted [19]. MCID: minimally clinically important difference, NA: no association. NRS: numerical rating score, VAS: visual analog scale.

**Table 3:** Functional Scores.

Outcomes	Mean	95% CI	I^2^	Included Study Groups (N)	Sample Size (N)	MCID Achieved
**Kinesio Taping Functional Scores**						
Baseline [24,25,28,32,34,35,36,37]	64.19	53.70–74.68	98%	9	140	NA
Follow-up 2-weeks [28,36]	86.21	78.45–93.98	97%	3	47	Yes
Follow-up 6-weeks [24,25,28,32,34,37]	82.80	79.87–85.73	58%	6	90	Yes
Combined Recent Follow-up [24,25,28,32,34,35,36,37]	84.23	79.44–89.01	95%	9	140	Yes
**McConnell Taping Functional Scores**						
Baseline [27,39]	68.02	65.76–70.28	0%	2	48	NA
Follow-up 6-weeks/Combined Recent Follow-up [27,39]	86.00	83.82–88.17	0%	2	48	Yes

An MCID of 8 points increased from baseline was accepted [19]. MCID: minimally clinically important difference, NA: no association.

## Data Availability

The data set used for this meta-analysis will be shared upon request from the study authors.

## Supplementary Materials

The following supporting information can be downloaded at: https://www.mdpi.com/article/10.3390/jcm13237476/s1, File S1: Actual search strategies; Table S1: Taping and exercise protocols; Figure S1: Kinesio taping pain scores; Figure S2: McConnell taping pain scores; Figure S3: Kinesio taping functional scores; Figure S4: McConnell taping functional scores.

## Abbreviations and Acronyms

AKPS	Anterior knee pain scale
CI	Confidence interval
MCID	Minimum clinically important difference
MD	Mean difference
NRS	Numeric pain rating scale
PFCA	Patellofemoral congruence angle
PFP	Patellofemoral pain
PRISMA	Preferred reporting items for systematic reviews and meta-analyses
ROBINS-I	Risk of bias in non-randomised studies of interventions
ROB2	Version 2 of the Cochrane risk-of-bias tool for randomized trials
VAS	Visual analog scale
