# Supplementary material for: Prolonged Taping with Exercise Therapy for Patellofemoral Pain in Adults: A Systematic Review and Single-Arm Meta-Analysis
Source: J Clin Med. 2024 Dec 9;13(23):7476. doi: 10.3390/jcm13237476 (PMC11641958; doi:10.3390/jcm13237476)

Supplemental Item S3: Forest plots of Kinesio and McConnell taping interventions

Supplementary Figure S1: Kinesio taping combined pain scores. N.B Agostini et al., 2023 and Arrebola et al., 2020 used the NRS, whilst all other articles used the VAS. A) baseline, B) 2-weeks, C) 4-weeks, D) 6-weeks, E) 12-weeks, F) combined recent follow-up. NRS: numerical rating score, VAS: visual analogue scale.

A)

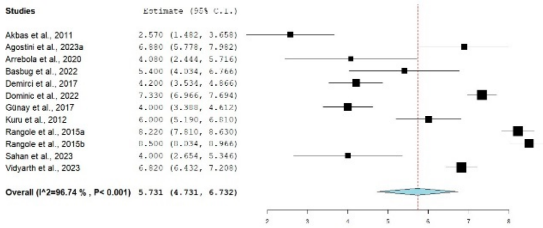

B)

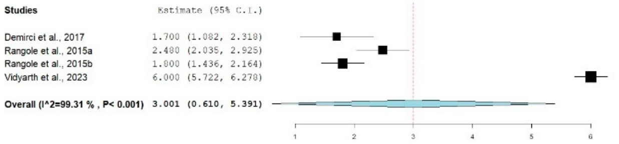

C)

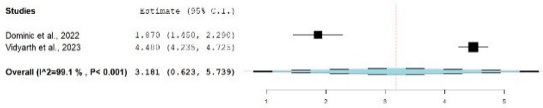

D)

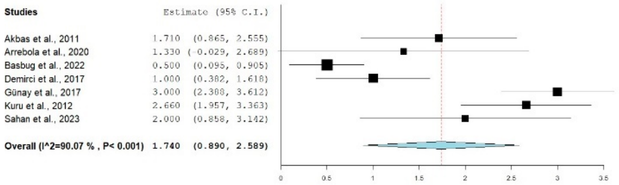

E)

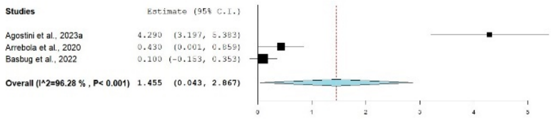

F)

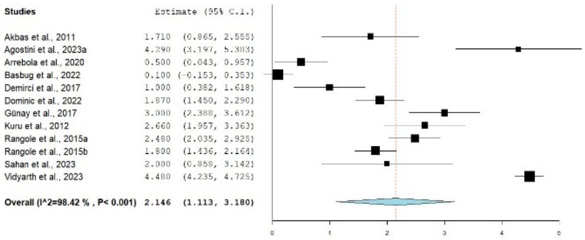

Supplementary Figure S2: McConnell taping combined pain scores. N.B Agostini et al., 2023 used the NRS, whilst all other articles used the VAS. A) baseline, B) 4-weeks, C) 6-weeks, D) combined recent follow-up. NRS: numerical rating score, VAS: visual analogue scale.

A)

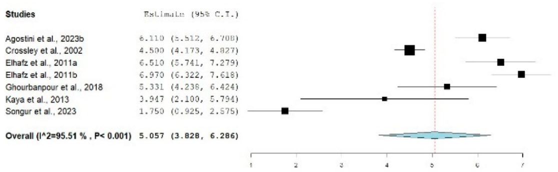

B)

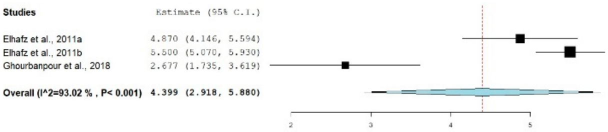

C)

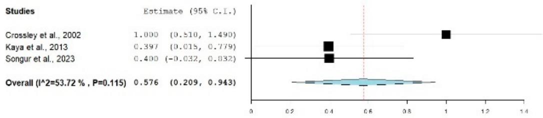

D)

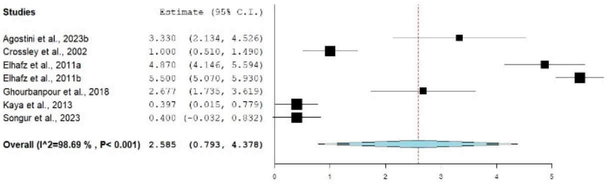

Supplementary Figure S3: Kinesio taping functional scores. A) baseline, B) 2-weeks, C) 6-weeks, D) combined recent follow-up.

A)

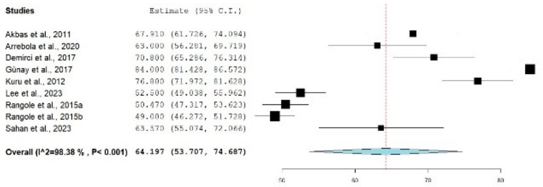

B)

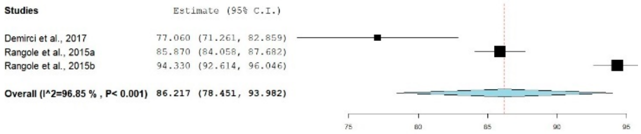

C)

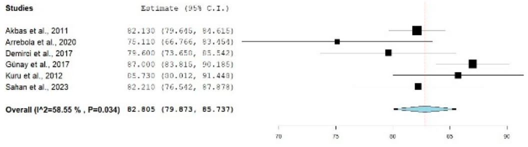

D)

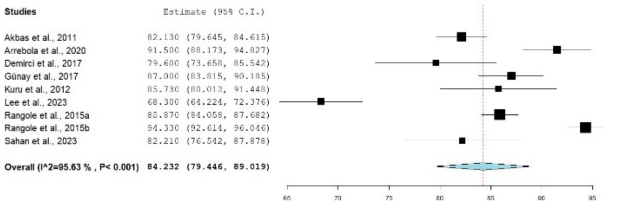

Supplementary Figure S4: McConnell taping functional scores. A) baseline, B) 6-weeks/ combined recent follow-up.

A)

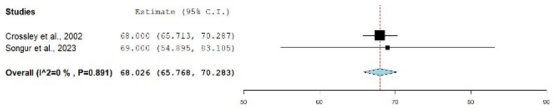

B)

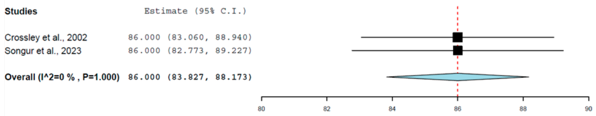

Supplement: Supplementary file 1 [file jcm-13-07476-s001.zip › Figue S1-S4.pdf]
