# Supplementary material for: Prolonged Taping with Exercise Therapy for Patellofemoral Pain in Adults: A Systematic Review and Single-Arm Meta-Analysis
Source: J Clin Med. 2024 Dec 9;13(23):7476. doi: 10.3390/jcm13237476 (PMC11641958; doi:10.3390/jcm13237476)
Supplement: Supplementary file 1 [file jcm-13-07476-s001.zip › File S1 ACTUAL SEARCH STRATEGIES.pdf]

SUPPLEMENTARY ITEM S1

Actual Search Strategies

Searches Performed for period 5/05/23-

Search date 28/06/24

PubMed

((((((Patellofemoral Pain Syndrome) OR (Anterior Knee Pain Syndrome)) OR (Patellofemoral Syndrome)) OR (Patellofemoral Pain)) OR (Patellofemoral Pains)) OR (anterior knee pain))

AND

(((((taping) OR (tape)) OR (strapping)) OR (kinesio)) OR (kinesiotape)) Sort by: **Publication Date**

("patellofemoral pain syndrome"[MeSH Terms] OR ("patellofemoral"[All Fields] AND "pain"[All Fields] AND "syndrome"[All Fields]) OR "patellofemoral pain syndrome"[All Fields] OR ("patellofemoral pain syndrome"[MeSH Terms] OR ("patellofemoral"[All Fields] AND "pain"[All Fields] AND "syndrome"[All Fields]) OR "patellofemoral pain syndrome"[All Fields] OR ("anterior"[All Fields] AND "knee"[All Fields] AND "pain"[All Fields] AND "syndrome"[All Fields]) OR "anterior knee pain syndrome"[All Fields]) OR ("patellofemoral pain syndrome"[MeSH Terms] OR ("patellofemoral"[All Fields] AND "pain"[All Fields] AND "syndrome"[All Fields]) OR "patellofemoral pain syndrome"[All Fields] OR ("patellofemoral"[All Fields] AND "syndrome"[All Fields]) OR "patellofemoral syndrome"[All Fields]) OR ("patellofemoral pain syndrome"[MeSH Terms] OR ("patellofemoral"[All Fields] AND "pain"[All Fields] AND "syndrome"[All Fields]) OR "patellofemoral pain syndrome"[All Fields] OR ("patellofemoral"[All Fields] AND "pain"[All Fields]) OR "patellofemoral pain"[All Fields]) OR ("patellofemoral pain syndrome"[MeSH Terms] OR ("patellofemoral"[All Fields] AND "pain"[All Fields] AND

"syndrome"[All Fields]) OR "patellofemoral pain syndrome"[All Fields] OR ("patellofemoral"[All Fields] AND "pains"[All Fields]) OR "patellofemoral pains"[All Fields]) OR (("anterior"[All Fields] OR "anteriores"[All Fields] OR "anteriorization"[All Fields] OR "anteriorized"[All Fields] OR "anteriors"[All Fields]) AND ("knee"[MeSH Terms] OR "knee"[All Fields] OR "knee joint"[MeSH Terms] OR ("knee"[All Fields] AND "joint"[All Fields]) OR "knee joint"[All Fields]) AND ("pain"[MeSH Terms] OR "pain"[All Fields]))) AND ("tape s"[All Fields] OR "taped"[All Fields] OR "tapes"[All Fields] OR "taping"[All Fields] OR "tapings"[All Fields] OR "tape"[All Fields] OR ("strap"[All Fields] OR "strapped"[All Fields] OR "strapping"[All Fields] OR "straps"[All Fields]) OR "kinesio"[All Fields] OR ("athletic tape"[MeSH Terms] OR ("athletic"[All Fields] AND "tape"[All Fields]) OR "athletic tape"[All Fields] OR "kinesiotape"[All Fields] OR "kinesiotaping"[All Fields]))

21

Embase

Session Results

....

No.	Query Results	Results	Date
#18.	#8 AND #16 AND [05-05-2023]/sd	47	27 Jun 2024
#17.	#8 AND #16	440	27 Jun 2024
#16.	#9 OR #10 OR #11 OR #12 OR #13 OR #14 OR #15	102,992	27 Jun 2024
#15.	'kinesiotape'/exp OR kinesiotape	210	27 Jun 2024
#14.	'kinesio'/exp OR kinesio	1,435	27 Jun 2024
#13.	strap*:ti,ab,kw	5,543	27 Jun 2024
#12.	strapping	611	27 Jun 2024

#11.	tape*:ti,ab,kw	89,447	27 Jun 2024
#10.	'tape'/exp OR tape	33,274	27 Jun 2024
#9.	'taping'/exp OR taping	3,758	27 Jun 2024
#8.	#1 OR #2 OR #3 OR #4 OR #5 OR #6 OR #7	15,309	27 Jun 2024
#7.	anterior AND knee AND pain	10,744	27 Jun 2024
#6.	'anterior knee pain'/exp OR 'anterior knee pain'	2,991	27 Jun 2024
#5.	'patellofemoral pains' OR (patellofemoral AND pains)	18	27 Jun 2024
#4.	'patellofemoral pain'/exp OR 'patellofemoral pain' OR (patellofemoral AND ('pain'/exp OR pain))	6,393	27 Jun 2024
#3.	'patellofemoral syndrome'/exp OR 'patellofemoral syndrome' OR (patellofemoral AND ('syndrome'/exp OR syndrome))	2,844	27 Jun 2024
#2.	'anterior knee pain syndrome' OR (anterior AND ('knee'/exp OR knee) AND ('pain'/exp OR pain) AND ('syndrome'/exp OR syndrome))	1,760	27 Jun 2024
#1.	'patellofemoral pain syndrome'/exp OR 'patellofemoral pain syndrome' OR (patellofemoral AND ('pain'/exp OR pain) AND ('syndrome'/exp OR syndrome))	2,666	27 Jun 2024

....

Cochrane

Search Name:

Date Run: 27/06/2024 15:54:51

Comment:

ID Search Hits

#1 MeSH descriptor: [Patellofemoral Pain Syndrome] explode all trees 375

#2 (patellofemoral pain syndrome):ti,ab,kw (Word variations have been searched) 763

#3 (anterior knee pain syndrome):ti,ab,kw (Word variations have been searched) 357

#4 (patellofemoral pain):ti,ab,kw (Word variations have been searched) 1187

#5 (patellofemoral pains):ti,ab,kw (Word variations have been searched) 1187

#6 (anterior knee pain):ti,ab,kw (Word variations have been searched) 2047

#7 {OR #1-#6} 2802

#8 (taping):ti,ab,kw (Word variations have been searched) 8179

#9 (tape*):ti,ab,kw (Word variations have been searched) 15064

#10 (strap*):ti,ab,kw (Word variations have been searched) 984

#11 {OR #8-#10} 15850

#12 #7 AND #11 287 limit to after 5/5/23 22

CINAHL

S1	(patellofemoral pain syndrome or patellofemoral or patellofemoral syndrome or anterior knee pain syndrome) OR patellofemoral syndrome OR patellofemoral pain OR Patellofemoral Pains OR anterior knee pain	5,929

S2	(taping or kinesio or kinesiotape or kinesiotaping) OR tape OR (strapping or strap or strapped)	9,703
S3	athletic tape	673
S4	(MM "Athletic Tape")	288
S5	S2 OR S3 OR S4	9,703
S6	S1 AND S5	282
S7	S6 2023-2024	12

Scopus

((TITLE-ABS-KEY(taping)) OR (TITLE-ABS-KEY(tape)) OR (TITLE-ABS-KEY(strapping)) OR (TITLE-ABS-KEY(kinesio)) OR (TITLE-ABS-KEY(kinesiotape))) AND ((TITLE-ABS-KEY(Patellofemoral Pain Syndrome)) OR (TITLE-ABS-KEY(Anterior Knee Pain Syndrome)) OR (TITLE-ABS-KEY(Patellofemoral Syndrome)) OR (TITLE-ABS-KEY(Patellofemoral Pain)) OR (TITLE-ABS-KEY(Patellofemoral Pains)) OR (TITLE-ABS-KEY(anterior knee pain))) 2023-2024
34

Web of Science

TS=((Patellofemoral Pain Syndrome) OR (Anterior Knee Pain Syndrome) OR (Patellofemoral Syndrome) OR (Patellofemoral Pain) OR (Patellofemoral Pains) OR (anterior knee pain)) 10,808

TS=(taping OR tape* OR strapping OR kinesio OR kinesiotape) 160,880

#1 AND #2 374
