# Supplementary material for: Prolonged Taping with Exercise Therapy for Patellofemoral Pain in Adults: A Systematic Review and Single-Arm Meta-Analysis
Source: J Clin Med. 2024 Dec 9;13(23):7476. doi: 10.3390/jcm13237476 (PMC11641958; doi:10.3390/jcm13237476)
Supplement: Supplementary file 1 [file jcm-13-07476-s001.zip › Table S1 TAPING AND EXERCISE PROTOCOLS.pdf]

SUPPLEMENTARY ITEM 2

Supplementary Table S1: Taping and exercise protocols

Studies	Taping Protocol	Exercise Protocol
Akbas et al., 2011	Individually designed with origin to insertion technique. Kinesio tape applied on VMO and quadriceps femoris for proprioceptive stimulation. In addition, it was applied to VL, ITB/TFL and hamstring muscles to relieve tightness.	Six-week home physiotherapy program with exercises added as needed. The program included:<br>-Stretching iliotibial band/ tensor fasciae latae (ITB/TFL) complex, hamstring, and quadriceps muscles<br>-Isometric and isotonic exercises for quadriceps, hip adductors, gluteus medius and maximus<br>-Open chain exercises such as straight leg raise, and leg raise with internal and external rotation<br>- Closed chain exercises such as mini squat.
Agostini et al., 2023a*	Kinesio taping was administered. The chosen application (the simplest one to reproduce at home by the patient in autonomy) consists of an inverted Y with an approximate length of 20-25 cm (depending on the anatomical characteristics of the patient) of 10-15% tension (without tension) for the stimulation of the muscle bellies of the quadriceps muscle (VM, VL).	Four strengthening exercises; 1) supine quadriceps isometry; 2) leg elevation to knee extended from supine; 3) seated knee extension; 4) squat up to 90° with TheraBand. Three stretching exercises: 1) prone quadriceps stretch; 2) supine hamstring stretch and 3) standing ilio-tibial band stretch. Exercises performed daily for 4 months.
Agostini et al., 2023b*	McConnel taping was administered involving the use of a rigid bandage to perform the medial glide manoeuvre (Strappal® was used), applied over a layer of neutral tension skin protection (Hypafix® was used) to avoid complications such as reactions allergies or friction between the skin and the rigid bandage. Both before and after applying the skin protector, the subjects were taught how to draw the edges of the patella to facilitate the execution of the bandage	Four strengthening exercises; 1) supine quadriceps isometry; 2) leg elevation to knee extended from supine; 3) seated knee extension; 4) squat up to 90° with TheraBand. Three stretching exercises: 1) prone quadriceps stretch; 2) supine hamstring stretch and 3) standing ilio-tibial band stretch. Exercises performed daily for 4 months.
Arrebola et al., 2020	Taping was performed with anchorage on suprapatellar region with end anchorage on tibial tuberosity with 0% tension (therapeutic zone had >50% tension on lateral region). Taping was applied once weekly throughout 12-weeks by a physiotherapist.	Weeks 1-4: strengthening exercises (80% 1RM): hip abductors, quadriceps in closed kinetic chain, and triceps surae (3 sets of 12 repetitions), strengthening with elastic resistance: quadriceps in open kinetic chain and lateral hip rotators (3 sets of 12 repetitions each)

		Weeks 4-8: same as prior weeks plus motor control exercises and core training (3 sets of 30s each of planks and lateral planks)<br>Weeks 8-12: same as prior weeks plus progression of the motor control exercises to unstable planes and core training (3 sets of 1 min each of planks and lateral planks).
Basbug et al., 2022	Patellar + ankle correction taping: mechanical correction tape was applied for the knee and foot. 'I' taping for accurate positioning of the tissue was applied to the patients to neutralize the patellofemoral joint.	Progressive home neuromuscular exercise program for knee/hip stabilization along with a weekly researcher physiotherapist-controlled compliance.
Crossley et al., 2002	Taping was applied in order of anterior tilt, medial tilt, glide, and fat pad unloading. The patients were taught to independently apply tape and wear tape during all waking hours.	Individual sessions lasting 30 to 60 minutes, once weekly for 6 weeks involved retraining of vastus medialis oblique, gluteal muscle strengthening exercises, and stretching of soft tissue. Furthermore, a daily home program was given to be done twice daily.
Demirci et al., 2017	A Y'-shaped kinesio tape applied using the muscle technique. 2 pieces of 'I'-shaped tape were stretched by 75% through the mechanical correction technique and applied around patellar circumference.	Home exercise program: hamstring muscle stretching (8-10 reps of 20s hold), straight leg raise (3 sets 10 reps), bridge exercise (3 sets 10 reps), clam shell exercise for gluteus medius (3 sets 10 reps), 4-way-hip strengthening exercises with elastic bands (2 sets 10 reps), terminal knee extension with elastic band while patients were in standing position (2 sets 10reps), and mini-squatting exercises (2 sets 10 reps). Patients were asked to do these exercises in 3 sets a day along with 10 repetitions for a period of 6 weeks.
Dominic et al., 2022	Two 'Y 'cut Kinesio strips were taken. First strip applied on the superior patella to the tip of the inferior pole out of the fat pad. Second strip applied at the tibial tuberosity and directed out wide to the medial knee joint. The soft tissue was lifted towards the patella. The final tape was applied to the tibial tuberosity to go wide to the lateral joint line.	The protocol included a corrective exercise program (CEP) which consists of strengthening exercises for hip (abductors and external rotators), knee (extensors), and neuromuscular exercises.<br>First 3 weeks involved side lying hip abduction, knee extension, squats, lateral band walking, forward lunges and eccentric adduction of hip using medium level resistance loop bands. Last week involved one leg

		balance with knee flexion and one leg squats in addition to previous set of exercises.
Elhafz et al., 2011a**	Medial patellar taping was applied before exercise. Patients were instructed to maintain tape between sessions.	Open kinetic chain exercises including supine straight leg raise, supine isometric quadriceps exercise, and seated knee extension.
Elhafz et al., 2011b**	Medial patellar taping was applied before exercise. Patients were instructed to maintain tape between sessions.	Closed kinetic chain exercises including leg press, mini squats, squat-to-stand, stand-to-squat, and forward step-up exercise on stairs.
Ghourbanpour et al., 2018	The McConnel taping method was used such that the tape was maintained between treatments.	Strengthening exercises for quadriceps muscles with emphasis on vastus medialis oblique, closed chain exercises, stretching exercises for hamstring muscles and iliotibial band and patellar mobilization.
Günay et al., 2017	Vastus medialis obliquus facilitation and patellar functional correction.	Stretching (quadriceps, hamstring, gastrocnemius, and iliotibial band) and strengthening (quadriceps, gluteus medius) exercises twice weekly for 6 weeks with physiotherapist. Patients were encouraged to perform daily home exercises. Exercises were three sets with 10 repetitions.
Kaya et al., 2013	Sub-tape was first applied then the corrective tape was applied after. The taping applied for anterior tilt, medial glide, medial tilt, and unloading the fat pad until the patient's pain was reduced at least 50%.	Neuromuscular retraining exercises included isometric quadriceps exercises in sitting, straight leg raise exercises (neutral position) with ankle weights, terminal knee extension exercises with ankle weights, wall squats with ball between the knees, split squats with Theraband® Stability Trainer (blue colour), stepdown exercises (backward, forward and sideway), and single-leg balance exercises in different knee angle with Theraband® Stability Trainer (blue colour). Stretching included quadriceps, iliotibial band, hamstrings, and gastrocnemius muscles.
Kuru et al., 2012	2-inch I strip of Kinesio® Tex Tape split to a Y was used proximal to the superior patellar border. The separate 2-inch Y-shaped tape was used for VMO. The I strip was applied to VMO.	The sessions included strengthening and stretching of quadriceps, hamstrings, iliotibial tract, and gastrocnemius. They were individual and lasted for 45 to 50 minutes, 3-times a week for 6 weeks. Patients were asked to do exercises at home on nontreatment days. Compliance was monitored via a daily log.

Lee et al., 2023	The taping was wrapped around the patella along the quadriceps tendon and muscle to ensure patellar protection and stabilization.	The program included:<br>-Open kinetic-chain exercises including multi-directional straight leg raise and knee extension with knee adduction.<br>-Closed kinetic-chain exercises, including wall squat exercise with knee adduction and single-leg squat.<br>-Hip and core muscle strengthening was also included.<br>The exercises were carried out at home twice a day.
Rangole et al., 2015a**	Taping was applied to the quadriceps femoris for proprioceptive stimulation and to the hamstring to relieve tightness. The 'Y' strip was used in both techniques.	Patients were instructed to train at 60% of their maximum. A new 10-repetition maximum was established at the end of a week of training. Each exercise was repeated 3 sets of 10 repetitions with 1 minute rest after each set.<br>In the open kinetic chain exercise protocol, each exercise was held isometrically for a count of 6 seconds with a 3- second rest between repetitions. The subjects were instructed to perform the conventional static quadriceps, hamstring, and gastrocnemius muscle stretching exercises after each training session. All subjects were instructed to perform three repetitions of a 30-second static stretch of exercised muscle groups. The subjects were made to do one session under supervision when they visit in OPD for every alternative day and advised to do exercises at home once in a day. The open kinetic chain exercise program consisted of: 1) maximal static quadriceps muscle contractions (quadriceps muscle setting) with the knee in full extension, 2) straight-leg raises with the patient supine, 3) short arc movements from 10° of knee flexion to terminal extension, and 4) leg adduction exercises in the lateral decubitus position.
Rangole et al., 2015b**	Taping was applied to the quadriceps femoris for proprioceptive stimulation and to the hamstring to relieve tightness. The 'Y' strip was used in both techniques.	Patients were instructed to train at 60% of their maximum. A new 10-repetition maximum was established at the end of a week of training. Each exercise was repeated 3 sets of 10 repetitions with 1 rest

		minute after each set. Each exercise in the closed kinetic chain protocol was performed dynamically with a 3-second rest between repetitions. The subjects were instructed to perform the conventional static quadriceps, hamstring, and gastrocnemius muscle stretching exercises after each training session. All subjects were instructed to perform three repetitions of a 30-second static stretch of exercised muscle groups. The subjects were made to do one session under supervision when they visit in OPD for every alternative day and advised to do exercises at home once in a day. The closed kinetic chain exercise program consisted of: 1) Semi squat and seated leg presses, 2) one-third knee bends on one leg and on both legs, 3) stationary bicycling, 4) rowing-machine exercises, 5) step-up and step-down exercises, and 6) progressive jumping exercises.
Şahan et al., 2023	Patellar star taping was applied in the treatment group in which a physiotherapist applies 4 equal I-shaped bands with rounded edges and a gap in the middle for four-way stabilization.	All patients were given home exercises. These were first performed under the supervision of the physiotherapist and then the patients were instructed to do the exercises by themselves at home. The home-based exercises consisted of stretching (hamstring, gastrosoleus, TFL, and lumbar extensors), knee stabilization, and strengthening exercises (quadriceps, glutaeus maximus, medius, and VMO).
Songur et al., 2023	Hypoallergenic tape applied transversely to the anterior knee with athletic tape applied on lateral edge of the patella terminating on the medial femoral condyle.	Stretching and mobilization for knee and hip combined with progressive strengthening of knee extension, hip abduction and external rotations, balance exercises, core exercises, walking and running. Strengthening increased from mild to high every 2 weeks with all patients given a daily home exercise program.
Vidyarth et al., 2023	Two 6-inch strips applied at 0% elongation, two 7.5-inch strips at 25% elongation and two 9-inch strips at 50% of elongation in Y shaped technique. Applied to the lateral and medial side of the patella.	Unspecified static and dynamic quadriceps exercises.

*Are the same study with the Kinesio and McConnell groups treated as separate groups for this meta-analysis. **Are the same study with the open kinetic chain and closed kinetic chain groups treated as separate groups for this meta-analysis.
